# Precocious Puberty Associated with Testicular Hormone-secreting Leydig Cell Tumor

**DOI:** 10.7759/cureus.6441

**Published:** 2019-12-22

**Authors:** Eid Alagha, Shahd E Kafi, Mohamed Abdelmaksoud Shazly, Abdulmoein Al-Agha

**Affiliations:** 1 Pediatrics, King Abdulaziz University Hospital, Jeddah, SAU

**Keywords:** leydig cell tumor, pseudo precocious puberty, testicular tumors, male children

## Abstract

Leydig cell tumors (LCTs) are rare testicular tumors that may be a cause of precocious puberty in males. We present a 5-year-old boy with a five-month history of pubic hair appearance associated with an increase in penile length, scrotal hyperpigmentation, change in body odor, and bone age advanced by two years. His hormonal tests revealed the diagnosis of pseudo precocious puberty. Testicular ultrasound showed a unilateral right testicular enlargement. Surgery was performed to remove the mass. Histopathology confirmed the diagnosis of LCT. This case report highlights the importance of consideration of testicular tumors in boys with precocious puberty.

## Introduction

Leydig cell tumors (LCTs) are a sex cord-stromal gonadal tumor which arise from Leydig cells [1]. They are rare tumors with an incidence of 1%-3% of all testicular tumors [2] and represent only 1% of all solid tumors affecting children. There are 0.5-2 reported testicular tumors per 100,000 children and adolescents [3]. LCTs occur in about 3%-6% of pre-pubertal boys. However, they are considered the most common hormonal secreting testicular tumors [4]. A majority of pediatric LCTs present between the ages of five to 10 years, and the clinical picture mainly consists of pseudo-precocious puberty symptoms [5]. Although they are mostly unilateral, bilateral tumors occur in 3%-10% of the cases [6]. LCT is usually considered a benign pediatric tumor, but malignancy is seen in 10% of the reported cases. Ultrasonography has an important role in evaluating patients with suspected testicular tumors [7]. Surgical removal of the mass is curative in LCT and is usually associated with regression of puberty signs [8]. This case is of a 5-year-old boy who presented with precocious puberty resulting from an LCT.

## Case presentation

**Figure 1:**
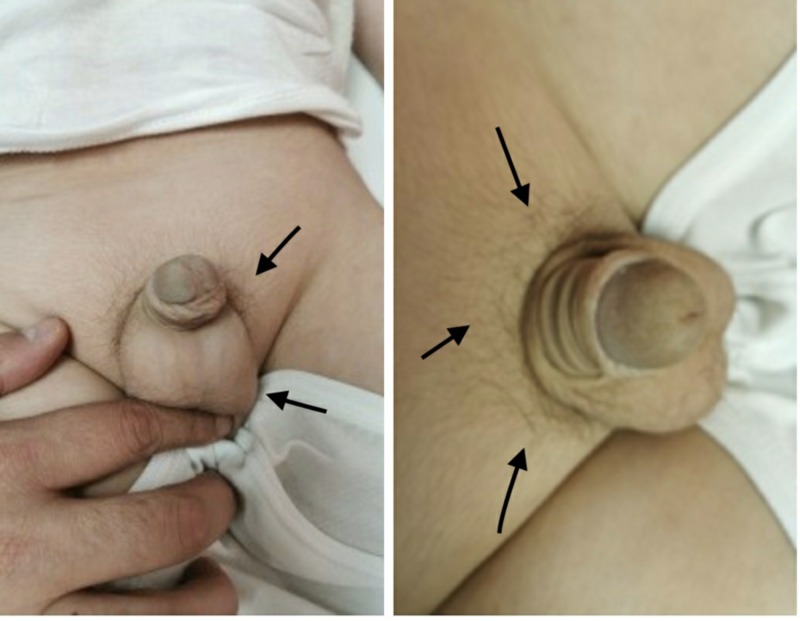
Pubic hair appearance and genital development (Tanner stage II - arrows)

**Figure 2:**
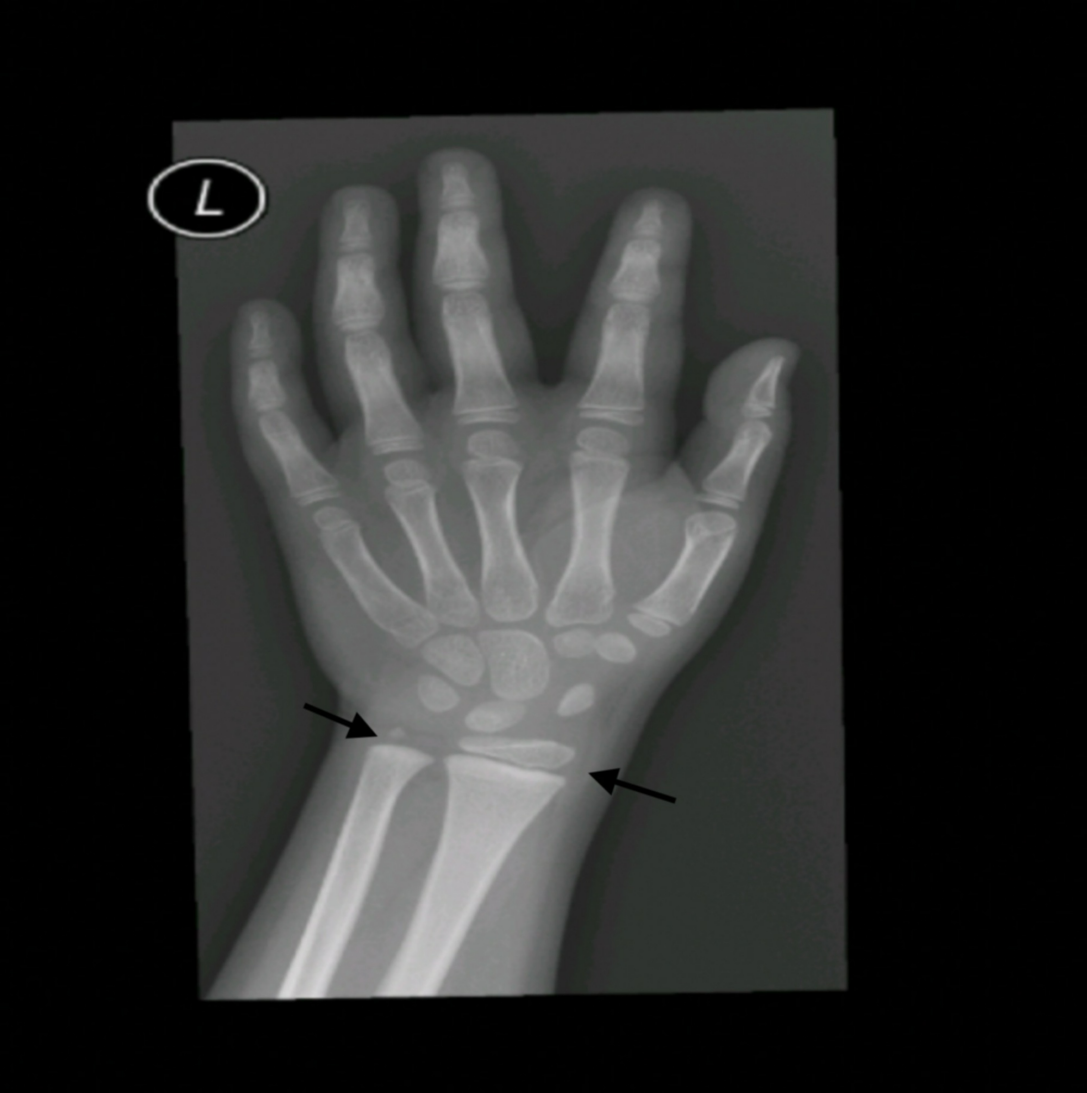
Bone age imagining reveals two-year advanced age

**Figure 3:**
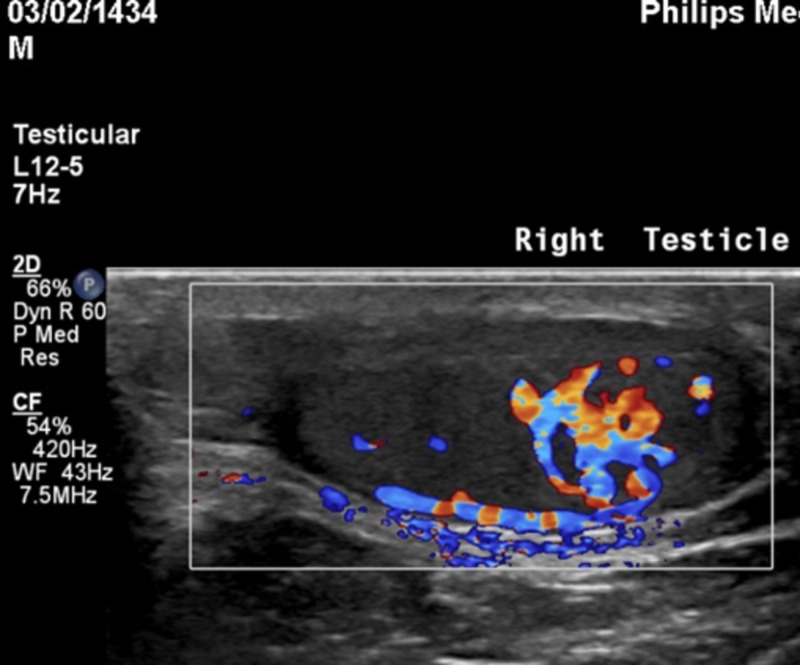
Testicular ultrasound shows right testicular mass

A 5-year-old boy was brought to the pediatric endocrinology clinic with a five-month history of pubic hair appearance associated with aggressiveness, increased penile length, and change in body odor. He was a product of full-term, normal vaginal delivery, with uneventful medical and surgical histories. There was no family history of endocrine tumors or precocious puberty. On examination, his vitals were unremarkable. Pubic hair and genitalia were Tanner stage II and the volume of each testis was 4 ml (Figure 1). Hormonal assay results are shown in Table 1. The gonadotropin-releasing hormone (GnRH) stimulation test showed a high testosterone level but no luteinizing hormone (LH) peak response. A two-year advanced bone age, together with laboratory investigations confirmed the diagnosis of pseudo precocious puberty (Figure 2). Non-classical congenital adrenal hyperplasia (NCAH) was excluded by an adrenocorticotropic hormone (ACTH) stimulation test (Table 2). Testicular Doppler ultrasound (US) revealed a unilateral enlargement of the right testis. Surgical removal of the tumor was performed. Histopathology confirmed the diagnosis of LCT (Figure 3).

**Table 1:** Hormonal assays including GnRH stimulation test confirming the diagnosis of pseudo precocious puberty GnRH: gonadotropin-releasing hormone.

Test	time	Result
Follicular stimulating hormone	0 min	0.2
	15 min	1.8
	30 min	2.5
	45 min	3.1
	60 min	4
	90 min	4.6
	120 min	5.1
Luteinizing hormone	0 min	0.2
	15 min	0.5
	30 min	0.7
	45 min	0.8
	60 min	0.9
	90 min	1
	120 min	1
Testosterone		7.7
		7.6

**Table 2:** Synacthen stimulation test excluding possibility of non-classical congenital adrenal hyperplasia DHEA: dehydroepiandrosterone sulfate; 17-OH-progesterone: 17-hydroxyprogesterone; ACTH: adrenocorticotropic hormone.

Hormone	0 min	30 min	60 min
17 OH progesterone	2.9 ng/ml	3.6 ng/ml	3.9 ng/ml
cortisol	348.6 nmol/l	734nmol/l	859.4 nmol/l
DHEA-S	0.54 umol/l	-	-
ACTH	2.12 pmol/l	-	-

## Discussion

This case demonstrates a rare instance of precocious pseudo puberty caused by an LCT. While testicular tumors in adults are a widely discussed topic, there is a significant shortage of data about prepubertal testicular tumors. Testicular tumors are rare in pediatric patients and account for only 1% of solid tumors among this age group [9]. Prepubescent testicular tumors have unique characteristics that distinguish them from their counterparts among adults and post-pubertal adolescents, including clinical presentation, epidemiology, and prevalence of histologic types [10-11].

Testicular tumors are subdivided into germ-cell tumors (teratoma, epidermoid cyst, and yolk sac tumors) and sex-cord stromal cell tumors (Sertoli-cell, Leydig-cell, and juvenile-granulosa cell tumors) [11]. LCTs with the active secretion of testosterone represent only 1%-3% of all testicular tumors. A majority of these tumors occur in middle-aged men with less than one-quarter of the cases in prepubertal children [12]. Most pediatric cases present with pseudo precocious puberty with a palpable mass in the testes, high testosterone, and low gonadotropin levels [13]. LCTs account for 10% of all cases of pseudo precocious puberty, with gynecomastia occurring in 10%-15% of these cases [14]. Ultrasound of the scrotum plays a major role in the diagnosis of LCT and can show the echogenicity of the mass and the degree of differentiation in comparison to surrounding tissues [15]. The conventional treatment for LCT is inguinal radical orchiectomy with lymphadenectomy if associated regional lymph nodes are involved. However, emerging evidence shows promising results using testes-preserving surgery with outcomes comparable to orchiectomy in prepubertal children [12-13,15].

Precocious puberty in boys is defined as the presence of testicular enlargement, penile growth, or pubic hair before the age of nine. Causes of precocious puberty can be categorized into two distinctive entities; central, or “true” precocious puberty, which involves an increase in GnRH, and peripheral, or “pseudo” precocious puberty, which occurs in a setting of low levels of GnRH. Central precocious puberty involves premature activation of the hypothalamic-pituitary-gonadal axis, and interaction between the central nervous system and various endocrine glands yields a sequential pattern of development of secondary sexual characteristics which is most often accompanied by a growth spurt [16]. In pseudo precocious puberty, changes are mediated by endogenous or exogenous sex steroid hormones independent of GnRH stimulation. There is a broad spectrum of congenital and acquired etiologies that can cause pseudo precocious puberty. Congenital adrenal hyperplasia is by far the most common congenital cause; other less common causes include McCune-Albright syndrome (MAS) and familial male-limited precocious puberty (FMPP). Acquired causes include adrenal, testicular, and rarely, human chorionic gonadotropin (HCG) or LH-secreting tumors in boys [17-18].

A literature review revealed several similar cases of prepubertal boys with LCTs presenting with precocious pseudo puberty manifested by increased penile length and various signs of androgenization [19-20]. Méndez-Gallart et al. stressed the crucial role of scrotal ultrasound in the diagnosis of precocious pseudo puberty in the absence of a palpable testicular mass [15]. Lignitz et al. suggested early treatment with GnRH following orchiectomy owing to the common presentation of central precocious puberty after the treatment of LCT [19].

Although rare, LCT is an important cause of precocious pseudo puberty in children. Testicular ultrasound can aid in early diagnosis and treatment in order to decrease the psychological and somatic impact of precocious puberty on prepubertal children.

## Conclusions

Although rare, LCT is an important cause of precocious pseudo puberty in children. Testicular ultrasound can aid in early diagnosis and treatment in order to decrease the psychological and somatic impact of precocious puberty on prepubertal children.
